# Child and Caregiver Reporting on Child Maltreatment and Mental Health in the Philippines Before and After an International Child Development Program (ICDP) Parenting Intervention

**DOI:** 10.1007/s40653-022-00483-0

**Published:** 2022-09-03

**Authors:** Emil Graff Ramsli, Ane-Marthe Solheim Skar, Vilde Skylstad, Disa Sjöblom, Zenona Gread, Wayomi Chiong, Ingunn Marie S. Engebretsen

**Affiliations:** 1 Centre for International Health (CIH), Department of Global Public Health and Primary Care (IGS), Faculty of Medicine and Dentistry, University of Bergen, Bergen, Norway; 2 The Norwegian Centre for Violence and Traumatic Stress Studies (NKVTS), Oslo, Norway; 3 Save the Children Finland (SCF), Helsinki, Finland; 4 Save the Children Philippines (SCP), Manila, Philippines

**Keywords:** Parent–child agreement, Child maltreatment, International child development program

## Abstract

Child maltreatment is a serious problem affecting millions of children. Research on self-reporting of child maltreatment has shown a difference in reporting between caregivers and children. Increased understanding of this has implications for further evaluations of parenting programmes and assessment of violence and maltreatment. The purpose of this study was to explore caregiver-child reporting discrepancies on child maltreatment and emotional health before and after piloting of the International Child Development Program (ICDP) in the Philippines. Data was collected from caregivers and their children before and after caregiver participation in ICDP. Participants were selected from the Pantawid Pamilyang Pilipino Program in Leyte by Save the Children. Caregivers and children completed a questionnaire with some adapted items from the Conflict Tactics Scale Parent–Child version (CTSPC), some relevant complementary items on psychological aggression and items from the emotional problems subscale from the Strength and Difficulties Questionnaire (SDQ). Matching items, subscales and total count scores were compared using paired t-tests in STATA 14. Forty-six caregivers and 43 children aged from 5–13 years participated at baseline, and 44 caregivers and 42 children at endline. At baseline, children reported significantly more maltreatment than their caregivers. The groups reported similarly at baseline and endline on the items from the subscale on emotional problems. Both children and caregivers had lower scores on our harsh discipline scale at endline, indicating improved parenting strategies after the intervention. These results indicate a difference in reporting of child maltreatment between caregivers and children, with higher rates reported by the children before the intervention, but not after. This is important because it illustrates child and caregiver perspectives on maltreatment, and how they can differ. As such, our findings point towards a positive effect of ICDP on parenting.

## Background

Child maltreatment is a widespread, global phenomenon affecting the lives of millions of children all over the world (Stoltenborgh et al., 2015). Different types of child maltreatment have been associated with several psychosocial risks, such as posttraumatic stress disorder (Gardner et al., 2019), depression, alcohol use, reduced academic performance (Fry et al., 2018) and overweight (Hussey et al., 2006). In a meta-analysis on the long-term health consequences of child physical and emotional abuse and neglect, Norman et al. (2012) argued that awareness of these consequences should encourage improved identification of the children at risk, as well as the development of effective measures to protect children and prevent violence.

Several studies have shown a difference between children’s and caregivers’ self-report of parent to child maltreatment (Compier-de Block et al., 2017). Existing research by Kobulsky et al. on children who were recently investigated by the child protective services documented a low to moderate correspondence between parent and child reports of past-year child physical abuse (Kobulsky et al., 2017). Especially when it comes to physical abuse and violence, children reported more physical abuse than their caregivers did (Fung & Lau, 2010; Kobulsky et al., 2017). A recent study of 6653 Norwegian parent–child dyads referred to child and adolescent mental health services from 2012 to 2017, showed that children reported significantly more exposure to accidents or illness, community violence and sexual abuse than what their caregivers did, but with no difference on reporting of domestic violence. The screening questions were asked in one of the first visits to the clinic in the start of an intervention (Skar et al., 2021). Children’s perspective on child maltreatment compared to the perspective of caregivers can give valuable insight into the actual prevalence of maltreatment, as well as how behavior is perceived differently between children and adults. 

Meta-analyses on the efficacy of parenting programs on reducing violence against children has shown promising results. Vlahovicova et al. (2017) showed that maltreating parents that went through a parenting program had an eleven percentage point decrease in absolute risk of child maltreatment compared to maltreating parents that did not go through a parenting program. In another meta-analysis looking at parenting interventions in low- and middle income countries in East and South East Asia, results suggested that the interventions had an effect in reducing violence against children, as well as promoting positive interactions between the parent and the child (McCoy et al., 2020).

ICDP is an interactive program for caregivers focusing on communication, attachment, and positive disciplining (Hundeide & Rye, 2010). ICDP was developed by the Norwegian Professors Karsten Hundeide and Henning Rye with colleagues, and is recognized and used by organizations like UNICEF and the WHO. The underlying thought of the program is that the best way to help a child is to sensitize the child’s caregivers to the needs of the child (Hundeide & Rye, 2010). The ICDP was inspired by the attachment theories of Bowlby (Bowlby, 1969; Rye, 2001) and Ainsworth (1989), and also Trevarthen (1988) and Stern’s (2018) understanding of caregiver-child communication as an important factor for children’s emotional development and mental health, and Klein’s research on mediated learning (Klein, 2001; Rye, 2002). One of the pillars of the ICDP is to train facilitators from the community who can adapt the concepts to an existing child care system and then train the parents in the community (ICDP International Team, 2010). This training of caregivers to be facilitators, and then have them teach ICDP has been proven to lead to less maltreatment and a better home environment for the child to grow up in (Skar et al., 2015). Thus, the program may be particularly relevant both for identifying parent maltreatment and handling it by communicating and admitting this to self and counsellors.

In a systematic review looking at social determinants of health, Hunter and Flores (2021) found that poverty is associated with child maltreatment, and they also argued that screening for social determinants like poverty and housing instability could lead to referrals that again could prevent maltreatment. In a long-term perspective it is important to prevent social inequalities and poverty to decrease the prevalence of child maltreatment. More knowledge on the differences on how children and caregivers from low-income families report child maltreatment and child emotional health could therefore lead to better identification of maltreated children and prevention of further maltreatment.

This is an exploratory study with data on child maltreatment and emotional health from caregivers and their children from before and after a piloting of the International Child Development Program (ICDP), administered by Save the Children in the Philippines. The aim is to investigate any discrepancies between the two groups of informants, caregivers and their children, on child maltreatment and emotional health pre- and post-implementation of ICDP. We created a measure of negative discipline, which includes psychological aggression and violence in addition to physical child maltreatment. We did not include items about positive disciplining strategies (e.g., the caregiver explaining why something is wrong to the child) in the analysis. We have not identified previous studies examining differences in reporting of violence and maltreatment between children and caregivers before and after a parenting intervention. Examining this further could give important knowledge on the effect of parenting programs from the perspective of both children and caregivers, and was an important reason for why we wanted to study this further. In addition, understanding these differences more deeply could point out a direction for considering information and social desirability bias better, meaning that given large discrepancies, children’s perspectives should be assessed in parenting programmes.

It is of value to describe any difference between groups for increased understanding of the effect of the parenting intervention and the nature of reporting. However, there are always concerns related to interpretation of findings. No difference in outcome measures could reflect on the program delivery or limited behavior change in the parents. No difference at endline could also reflect on similar changes in the waiting list controls, reflecting some kind of waiting list bias. A difference in reporting of maltreatment could point towards a positive effect of the program delivery and changed parenting behavior. Errors in capturing and reporting are inherent challenges in behavioral research (Van den Broeck, 2013). Thus, a key value of this research is to provide a description of what kind of disciplining, both positive and negative, that is reported used within households.

## Methods

### Context and Population

In 2017 in the Philippines, Save the Children introduced the International Child Development Program (ICDP) in selected barangays (small administrative units or villages) in the Leyte province. This was part of a larger project focusing on making the Pantawid Pamilyang Pilipino Program more sensitive to development needs and rights of children (Engebretsen, 2018). The Pantawid program is a national poverty reduction program in the Philippines, and provides conditional cash transfers to poor households to improve health, nutrition and education (Kandpal et al., 2016). The ICDP parenting program was piloted prior to a planned national scale-up within the Pantawid Pamilyang Pilipino Program.

Eight barangays with a sufficient number of participants in the Pantawid Pamilyang Pilipino Program were purposively selected to be a part of the ICDP pilot in the study area. The study population included a subset of caregivers and their children from four randomly selected barangays out of the mentioned eight barangays. To be eligible for the study, the caregivers had to live in the selected barangays, provide oral informed consent, and have a child aged between 5–13 years, and be a part of the Pantawid Pamilyang Pilipino Program. All caregivers in the barangay that fulfilled these requirements were invited to participate. An informed consent form was read out laud due to a relatively high illiteracy rate. The children provided oral assent. They were informed they could withdraw at any time. There were no compensation or incentives given. The remaining pre-selected four barangays were put on a waiting list to receive the intervention later. The children were interviewed alone by a community worker known to the child which read the survey out loud to them.

### Study Design

The pilot intervention was an ICDP intervention that lasted four months and included weekly parenting sessions for the caregivers. Children did not receive any organized counselling or training sessions. Caregivers were divided in groups consisting of 8–12 participants. The sessions were led by 10 Save the Children collaborating community workers that were trained in the use of the ICDP the year prior to this program (2017). This study presented between- and within-group comparisons at baseline and endline. The groups that were compared were children and their caregivers before and after the intervention.

Caregivers and children were interviewed before and after the ICDP piloting. Out of 100 adults recruited from 8 barangays, 46 caregivers were included from the randomly chosen four barangays that participated in the intervention. Forty-three of their children answered at baseline, and 44 caregivers and 42 children answered at endline. Thirty-nine of the same children answered at both baseline and endline.

The baseline data collection was carried out between December 2017 and January 2018 and the endline data collection took place between August and September 2018. Data collection was done using Kobo Collect and data management using Open Data Kit (ODK) software.

### Measures

A questionnaire was made which included some items from the Strength and difficulties questionnaire (SDQ) and the Conflict tactics scale parent–child version (CTSPC) (Engebretsen, 2018), in addition to two culturally relevant items on child maltreatment previously used by Sherr et al. (2011). The items from the CTSPC and the two culturally relevant items were combined into a questionnaire to create a measure of harsh discipline and child maltreatment. The items that were included were items that were matching between the child and caregiver questionnaire. The items used are showed in Table 1. A consensus on the final tools were reached with input from Save the Children site representatives, community social-workers and the researchers. The tools were pretested in adjacent communities to the research sites and consensus on vernacular translation reached when language support was needed in addition to the English version which is widely spoken in the Philippines.

**Table 1:** Overview of items, subscales and answering alternatives included in the questionnaire

**Harsh discipline—CTSPC and related items– items grouped in the different subscales to caregivers and children** (Ch = Child; A = Adult)	
**Physical assault questions**	**Possible answers on each item in the CTSPC**
Ch: Used a stick, hairbrush, slipper (list cultural relevant objects) or other hard item to discipline you? A: Used a stick, hairbrush, slipper, belt or other hard item to discipline the child?	Never (0) Seldom (1) Sometimes (2) Always/often (3)
Ch: Slapped, punched or hit you on your head or face? A: Slapped, punched, pinched or hit the child on his/her head or face or ears?	
**Psychological aggression questions**	
Ch: Said you would be sent away or kicked out of the house? A: Said the child would be sent away or kicked out of the house	
Ch: Threatened to invoke ghosts or evil spirits, or harmful people? A: Threatened to invoke ghosts or evil spirits, or harmful people against the child?	
Ch: Insulted you by calling you dumb, lazy or other names like that? A: Called him or her dumb, lazy or other names like that?	
Ch: Withheld a meal to punish you?^a^ A: Withheld a meal to punish the child^a^	
Ch: Kept you out of school? ^a^ A: Kept the child out of school (as punishment)? ^a^	
**SDQ – emotional problems subscale**	
**Parent questions**	**Possible answers**
Is the child unhappy, downhearted or tearful?	Not true (0) Somewhat true (1) Certainly true (2)
Is nervous or clingy in new situations or easily loses confidence. (self-esteem)	
**Child questions/statements**	**Possible answers**
“I am sad”	Once in a while (0) Many times (1) All the time (2)
“I feel like crying”	
“Things bother me”	

^a^Items included based on formative work by Sherr et al. (2011)

CTSPC is used worldwide to measure both psychological and physical abuse of children by caregivers (Sierau et al., 2018). It includes the following subscales: non-violent discipline, psychological aggression and physical assault (minor, severe and very severe). CTSPC is designed to be administered to caregivers and children (Straus et al., 1998). CTSPC has been proven to be a scientifically and clinically sound tool for showing the child’s own perspective on psychological and physical abuse from an early age (Sierau et al., 2018). Questions included in the current study were about specific acts of aggression, and not about the child’s injury. Items and subscales from the CTSPC are listed in Table 1, and items not from the original scale are marked. From the CTSPC we included five items (e.g. if the respondent used a stick, hairbrush, slipper, belt or other hard item to discipline the child). In addition to the items from the CTSPC, we also included two culturally appropriate items on withholding a meal and keeping a child out of school as designed by Sherr et al. (2011) in our measure. The items from the original CTSPC were categorized into two subscales (physical assault and psychological aggression) in concordance with two of the subscales created by Straus et al. (1998) and the additional items were grouped into the psychological aggression subscale. We did not look at items related to non-violent discipline in this study. This left us with two subscales with the following items: two items about physical assault and five items about psychological aggression. Psychological aggression measures verbal or other symbolic acts done to induce fear in the child (Cotter et al., 2018). In addition, a scale consisting of all the seven items added together was created into a total added scale. Items values was not changed or turned since we only asked questions on negative discipline. We did not differentiate between minor, severe and very severe physical assault, because we only looked at two items related to assault.

SDQ, developed by the British psychiatrist Robert Goodman (Goodman, 1997), is a behavioral screening questionnaire about children from age 3–16, used to measure emotional problems, conduct problems, hyperactivity, peer relationship problems and prosocial behavior (Muris et al., 2003). SDQ has different versions for parents, teachers, and children. It has been translated to more than 80 languages. A review by Stone et al. showed that the psychometric properties of the SDQ were strong (Stone et al., 2010).

In the current study only items from the SDQ emotional problems subscale were included, and these items were compared between caregivers and children. The emotional problems subscale for the caregivers included two items, one was whether the child had shown depressive symptoms, and one about nervousness and clinginess in new situations. The child version of the same subscale included three items, one about feeling sad, one about frequency of crying, and one about if “things” bother the child or not (Table 1).

The items we are including in this study was part of a bigger questionnaire which also included socio-demographic information, in addition to questions about nutrition, gender expectations and several other subjects that we did not find relevant to this particular study. The questionnaire was translated and back translated from English to Cebuano and administered to the caregivers. Data collectors had face to face interviews with the participants. To capture self-reported ordinal quantification on a scale, items options such as “never”, “not so often”, “sometimes”, always/daily”, were supported by using a cup/drinking glass-illustration which corresponded to an increasing amount of water. For the CTSPC, the options were coded from 0–3, where 0 was never, 1 was seldom, 2 was sometimes and 3 was always/daily. The SDQ options were coded from 0–2. The response options of the SDQ items were different for the caregivers and the children. For the caregivers, the items were answered with not true (0), somewhat true (1), or certainly true (2), while the children got three alternative statements on the same items and had to choose which they found was most true. Because of the difference in how the scores were reported between the children and caregivers on the SDQ items, we have not done analytical comparisons between groups on the SDQ items, but rather chosen to include the item scores from that scale as descriptive information.

### Data Analysis

STATA 14 was used for analysis. Descriptive data were presented including means with standard deviations (SD) and 95% confidence intervals (95% CI). Medians and ranges were used to describe continuous variables. Categorical variables were presented with frequencies and percentages. Subscales of SDQ and CTSPC were constructed by combining the relevant variables together. Missing data was set to empty. Outliers were not considered as a huge problem after data entry, because data management including consistency checks were done. The data set was closely looked through, and actual outliers on both sides of the scale were chosen to still be included to give a better view of the prevalence of child maltreatment in the sample.

The children had their own questionnaire with many of the same items as the adults. How caregivers and children answered their questionnaire on the similar items, as specified in Table 1, was examined and reported both on item, subscale and total added scale levels. The analysis of similarities and discrepancies were done at both baseline and endline. Both a within-group comparison between baseline and endline for caregivers and children, respectively, and a between-group comparison between caregivers and children at baseline and endline were done. Items that did not fit or items that could only be found in one of the two questionnaires were excluded from the analysis. For an item to be considered as fitting to be part of this analysis it had to be about child maltreatment, disciplining strategies or child maltreatment, and also be present in similar way in both the child and caregiver questionnaire. For example, an item in the CTSPC that asked specifically about positive behavior was removed from the total added scale (if the child was explained why they did something wrong). It was removed because it did not fit into any of the three subscales that we compared within and between the groups. But because the item was a part of both the caregiver and child questionnaire this item has been reported separately in a descriptive manner in the results section. Other items, for instance the ones about nutrition, was cut from this analysis.

In addition, a table was made where the answers of the individual CTSPC items were grouped into two, either “never” or “ever”. If the answer on an item was “never”, it went into that group, but if the answer was either “not so often”, “sometimes” or “always/daily”, the answer was put into the “ever” category. This was done to see more clearly if the child had been exposed to maltreatment or not. The original authors of the CTSPC recommended creating a dichotomous prevalence variable in addition to a chronicity variable when looking at physical assault (Straus et al., 1998). Chan also used a dichotomous CTSPC-scale when looking at parent and child report of child maltreatment in Hong Kong (Chan, 2012). All the items asked the prevalence of maltreatment and harsh discipline within the last three months.

The values of the total added scales and sub-scales for children and adults were compared between groups and within groups using paired t-tests. We calculated mean differences within groups, and between groups, but chose paired t-tests as we wanted to present the mean differences within the caregiver-child dyads, not group level mean differences. Difference estimates are given as score differences with 95% confidence intervals (CI).

Checks for internal consistency were done and the Cronbach alpha coefficients for the child and adult items were calculated. The coefficient measured the internal consistency of the items from the CTSPC and the two culturally appropriately items added into the questionnaire for both caregivers and children, respectively. The Cronbach alpha for the child items was 0.76 at baseline and 0.57 at endline. For the caregivers the alpha was 0.40 at baseline and 0.60 at endline.

## Results

### Population Characteristics

The characteristics of the children is described in Table 2, and the caregiver characteristics are described in Table 3.

**Table 2:** Baseline characteristics of the children as reported by 46 of the caregivers in the piloting of the ICDP program, Leyte, Philippines 2018

***Categorical variables***	***Number, total* = *46***	***Percentage (%)***
Sex, child		
Boy	29	63
Girl	17	37
Caregiver’s relationship to child		
Mother	37	80.4
Father	3	6.5
Grandmother	6	13.0
Who the child is staying with		
Mother and father, incl. stepparents	37	80.4
Only mother	3	6.5
Grandparents	6	13.0
***Continuous variables***	***Mean***	***(95% CI)***
Age (n = 44)	10.7	(9.8;11.6)
Child’s highest level of education (0–9)		
Boys (n = 29)	4.9	(4.4;5.4)
Girls (n = 17)	3.9	(3.3;4.5)
Total (n = 46)	4.5	(4.1;4.9)
Number of siblings	3.7	(2.9;4.5)

**Table 3:** Baseline characteristics of the caregivers

*Categorical variables caregivers*	*Number, total* = *46*	*Percentage (%)*
Sex, caregiver		
Male	3	6.5
Female	43	93.5
Caregiver can read and write		
No	2	4.3
Yes	44	95.7
Education, caregiver		
Primary	36	78.3
Secondary or higher	10	21.7
Respondent’s marital status		
Married	39	84.8
Living together	5	10.9
Widowed	2	4.4
*Continuous variables*	*Mean*	*95% CI*
Years of education—caregivers (0–11) (n = 46)	5.6	(4.7;6.4)
Partner’s highest education, years (n = 28)	5.0	(3.9;6.1)
Number of people in the household (n = 46)	5.9	(5.2;6.6)

### Reporting on the Conflict Tactics Parent–child Scales

The CTSPC mean item scores from baseline and endline are shown in Table 4. There was a timespan of approximately seven months between the baseline questionnaire was answered until the ICDP intervention was finished and the respondents answered at endline. The item from the CTSPC with the largest decrease in frequency from baseline to endline was the item about disciplining by hitting the child with an item like a hairbrush or stick. The same could be observed among the children. (Table 5).

**Table 4:** Mean item scores with 95% CI of individual items of the Conflict Tactics Scale Parent and Child versions (CTSPC) and related items prior to (baseline (BL)) and after (endline (EL)) the piloting of the ICDP program in Leyte, Philippines, caregivers (adults) and children

CTSPC item and item summarized^a^	Adult baseline n = 46	Children baseline n = 43	Adult endline n = 44	Children endline n = 42
1. Used hard item to discipline child	0.8 (0.5;1.0)	1.2 (0.9;1.5)	0.1 (0.02;0.2)	0.4 (0.1;0.6)
2. Hit child on head/ears/face	0.2 (0.1;0.3)	0.4 (0.2;0.7)	0.1 (-0.0;0.2)	0.1 (-0.0;0.3)
3. Said would send away	0.2 (0.01;0.3)	0.3 (0.1;0.6)	0.3 (0.1;0.4)	0 (0;0)
4. Threatened to invoke ghosts/spirits/harmful people against child	0.5 (0.3;0.7)	0.7 (0.4;0.9)	0.3 (0.1;0.5)	0.3 (0.1;0.5)
5. Withheld meal/snack as punishment^b^	0.1 (-0.0;0.2)	0.3 (0.1;0.5)	0.1 (-0.1;0.1)	0.2 (-0.0;0.4)
6. Called child dumb/lazy/similar names	0.7 (0.4;0.9)	0.5 (0.2;0.7)	0.2 (0.0;0.3)	0.1 (-0.0;0.2)
8. Kept child out of school as punishment^b^	0	0.5 (0.3;0.7)	0.01 (-0.0;0.1)	0.1 (-0.0;0.2)

^a^Range for each item is 0–3. 0 = never, 1 = seldom, 2 = sometimes, 3 = always/often

^b^Items included based on formative work by Sherr et al. (2011)

**Table 5:** Items from the Conflict Tactics Scale Parent–Child version (CTSPC) and related items categorized into never or ever (seldom, sometimes, always/often) from caregivers (adults) and their children prior to (baseline (BL)) and after (endline (EL)) the piloting of the ICDP program in Leyte, Philippines

Item	Adult baseline n, %	Child baseline n, %	Adult endline n, %	Child endline n, %
1.Used a stick, hairbrush, slipper, belt or other hard item to discipline the child?				
Never	24 (52.2%)	14 (30.4%)	39 (84.8%)	33 (71.7%)
Ever	22 (48.8%)	29 (63.0%)	5 (10.9%)	9 (19.6%)
Missing	0	3 (6.5%)	2 (4.4%)	4 (8.7%)
2. Slapped, punched, pinched or hit the child on his/her head or face or ears?				
Never	38 (82.6%)	33 (71.7%)	42 (91.3%)	39 (84.8%)
Ever	8 (17.4%)	10 (21.7%)	2 (4.4%)	3 (6.5%)
Missing	0	3 (6.5%)	2 (4.4%)	4 (8.7%)
3. Said you would send him/her away or kick him/her out of the house?				
Never	38 (82.6%)	37 (80.4%)	36 (78.3%)	42 (91.3%)
Ever	8 (17.4%)	6 (13.0%)	8 (17.4%)	0
Missing	0	3 (6.5%)	2 (4.4%)	4 (8.7%)
4. Threatened to invoke ghosts or evil spirits, or harmful people against the child?				
Never	30 (65.2%)	27 (58.7%)	34 (73.9%)	33 (71.7%)
Ever	16 (34.8%)	16 (34.8%)	10 (21.7%)	9 (19.6%)
Missing	0	3 (6.5%)	2 (4.4%)	4 (8.7%)
5. Withheld a meal or allowance for the snacks to punish him or her?^a^				
Never	44 (95.7%)	36 (78.3%)	43 (93.5%)	39 (84.8%)
Ever	2 (4.4%)	7 (15.2%)	1 (2.2%)	3 (6.5%)
Missing	0	3 (6.5%)	2 (4.4%)	4 (8.7%)
6. Called him or her dumb, lazy or other names like that?				
Never	25 (54.4%)	31 (67.4%)	39 (84.8%)	39 (84.8%)
Ever	21 (45.7%)	12 (26.1%)	5 (10.1%)	3 (6.5%)
Missing	0	3 (6.5%)	2 (4.4%)	4 (8.7%)
8. Kept the child out of school (as punishment)?^a^				
Never	46 (100%)	26 (56.5%)	43 (93.5%)	40 (87.0%)
Ever	0	17 (28.3%)	1 (2.2%)	2 (4.4%)
Missing	0	3 (6.5%)	2 (4.4%)	4 (8.7%)

^a^Items included based on formative work by Sherr et al. (2011)

When looking at the reporting of the children and caregivers before and after the intervention, there was a statistically significant difference within groups between baseline and endline for both subscales of the CTSPC, and for the CTSPC total scale (Table 6). The differences were significant for both children and adults. In both subscales of the CTSPC we saw that both children and caregivers reported lower incidence of physical assault and psychological aggression after the intervention (Table 6).

**Table 6:** Means and mean differences (MD) with 95% CI for the Conflict Tactics Scale Parent–Child version (CTSPC) and Strengths and Difficulties questionnaire (SDQ) sub-scales and total scores prior to (baseline (BL)) and after (endline (EL)) the piloting of the ICDP program in Leyte, Philippines. Caregivers (adults) and children

	Means				MD between groups		MD within groups	
CTSPC subscales	Adult, BL n = 46	Child, BL n = 43	Adult, EL n = 44	Child, EL n = 42	Child–adult BL n = 43	Child–adult EL n = 40	Children BL-EL n = 39	Adults BL-EL n = 44
Physical assault, r 0–6	1.0 (0.7;1.3)	1.7 (1.2;2.1)	0.2 (0.0;0.3)	0.5 (0.2;0.9)	0.7 (0.2;1.2)*	0.4 (-0.1;0.8)	1.1 (0.6;1.6)*	0.8 (0.5;1.2)*
Psychological aggression, r 0–15	1.5 (1.0;1.9)	2.2 (1.3;3.0)	0.8 (0.4;1.2)	0.7 (0.3;1.1)	0.7 (-0.2;0.1.6)	-0.1 (-0.7;0.0)	1.5 (0.8;2.3)*	0.7 (0.1;1.3)*
Total, r 0–21	2.5 (1.9;3.1)	3.8 (2.6;5.0)	1.0 (0.5;1.4)	1.1 (0.5;1.8)	1.4 (0.2;2.6)*	0.2 (-0.5;1.0)	2.7 (1.7;3.7)*	1.5 (0.8;2.2)*
SDQ subscale	Means						MD within groups	
Emotional problems, r 0–4	0.6 (0.4;0.8)	0.9(0.5;1.3)	0.5(0.3;0.8)	0.6 (0.3;0.9)			0.3 (-0.2;0.8)	0.1 (-0.3;0.4)

*r* range

* significant difference

A significant difference was found at baseline between children and caregivers on the total value of the CTSPC. This indicates that children reported significantly more caregiver to child-maltreatment than the adults did. Looking at the subscales of CTSPC at baseline, there was also a significant mean score difference between children and caregivers when reporting on physical assault where the children reported more violence than what the caregivers did. There was not a statistically significant score difference between children and caregivers when reporting on the psychological aggression subscale at baseline, and on this subscale the caregivers reported slightly more psychological aggression than the children did at endline, however, the numbers were very similar and not statistically significant.

At endline there was not a statistically significant difference between children and caregivers on the total value of CTSPC or any of the subscales.

The item that was excluded from the total added scale asked about if the parent explained to the child why something was wrong. At baseline there was a significant difference between caregivers and children on this item. (Caregivers reported that they explained more than the children reported that they did. At endline there was not a significant difference between groups on this item, and there was not a significant difference within groups when comparing baseline and endline.

### Emotional Health Reported in the Child and Caregiver Versions of the Strengths and Difficulties Questionnaire

In the emotional health subscale of the SDQ, participants reported if the child showed signs of emotional problems.

When comparing the results within groups at baseline and endline, there was not found a significant score difference on child emotional health, neither in child reports or in caregiver reports.

## Discussion

In this study we investigated the changes in reporting of child maltreatment and emotional problems before and after the caregivers participated in a pilot of the ICDP program, prior to a planned scale-up of the ICDP in the Philippines by Save the Children. We reported on whether there were discrepancies between child- and caregiver reporting on the same items, subscales and items regarding parenting maltreatment practices and children’s emotional health.

Physical assault and psychological aggression were highly present in the study population at baseline, where 49% of the caregivers and 63% of children reported that children were hit with an object and 35% of both children and caregivers reported that the child had been threatened by the caregiver within the last three months. Degrading language was also practiced by almost half (46%) of the caregivers according to the children, and 26% according to the caregivers. All these behaviors were reduced after the ICDP intervention, for example after the intervention 11% of caregivers and 20% percent of children reported the child being hit with an object within the last three months. Ten percent of the caregivers and seven percent of the children reported use of any degrading language against the child at endline. This means that both children and caregivers reported lower scores on the harsh discipline items and subscales. The changes in reporting on the items may be an indicator that the ICDP intervention influenced disciplining strategy, in particular physical assault. A study from an ICDP intervention in Mozambique also showed a changed pattern in physical disciplining with a decrease in hitting and harsh discipline after the intervention (Skar et al., 2014). It is important to consider that the interviews at endline were done shortly after the intervention, so we do not know about its long-term effects. The interviews at endline were done the same month or the month after the intervention concluded. It has been shown by Skar et al. (2015) that key positive effects of the ICDP can be sustained over some time (6–12 months post-intervention), but at a somewhat lower level.

Our findings of positive effects of the ICDP intervention harmonizes with other studies on ICDP in other settings, including in population samples in Norway (Sherr et al., 2014; Skar et al., 2015, 2017). As previously mentioned, the aim of the ICDP is to sensitize parents and caregivers about positive limit setting, communication, and attachment. A meta-analysis by Stith et al. (Stith et al., 2009) on the risk factors in child maltreatment found that the quality of the parent–child relationship was a factor strongly related to neglect, and moderately related to physical abuse. This research may as such support the aim and course content in the ICDP related to positive parenting.

The change in reporting of maltreatment from baseline to endline points to a positive effect of the intervention. A possible explanation is that the caregivers learned about positive disciplining strategies in the ICDP course, and maybe recognized behavior and disciplining strategies in their daily life that they changed after the intervention. It is also possible that the caregivers learned what was the goal of the program, and changed their answers to avoid negative consequences, as mentioned previously. But since the children reported a change, that strengthens the evidence for an effect of the program.

In a systematic review and meta-analysis on parenting interventions in low- and middle-income countries, McCoy and colleagues(McCoy et al., 2020) showed that a parenting intervention can reduce rates of child physical and psychological abuse, as well as promote positive interactions between parent and child. The study by McCoy and colleagues did not examine any interventions using ICDP, but other programs with a similar aim of instructing caregivers on positive parenting. This shows similar effects to the ICDP intervention in this study.

There was only a slight non-significant difference in how the children and caregivers reported on child emotional problems on the emotional problem items before and after the intervention. A paper on the discrepancy of parent–child reporting of emotional and behavioral problems in Norwegian children aged 10–13 years showed that children self-reported significantly more symptoms of emotional problems than their parents did (Van Roy et al., 2010). The reason for the small difference in our study for the SDQ items could be because the emotional health assessment had a different structure between the children and adults. In the questionnaires in this study, the children chose which of three statements that fit them the best on different items, while the caregivers´ answered statements about the children with answers on how true they were. In contrast, the CTSPC items in our study was worded much more similarly between adults and children. Because of this substantial difference in the design of the questionnaire between the two groups we did not analytically compare the two groups, and only report descriptively on the differences within groups.

It is common that caregivers report lower rates of child maltreatment than children (Chan, 2012; Kobulsky et al., 2017; Kolko et al., 1996). One possible explanation for underreporting of child maltreatment from caregivers is fear of negative consequences afterwards (Compier-de Block et al., 2017), such as being reported to the child protective services or the police. But children can also be biased and report in a certain way because of embarrassment over the maltreatment, or have a wish to protect their parents or caregivers (Compier-de Block et al., 2017). The results may have been affected by the young age of the children responding to the questionnaire who were between 5–13 years, with the mean age being 11, because when looking at parent–child agreement on child maltreatment, Compier de-Block et al. found in a multi-generational study that older children and youth reported more maltreatment than younger children (Compier-de Block et al., 2017). Older children and youth in the study by Compier de-Block were over 18 years old and answered on items related to their experiences of maltreatment in childhood. It was hypothesized that the difference in reporting of maltreatment could be because of older children may have realized with age that their caregivers’ behavior was not normative, and then report more maltreatment when asked about it. Our study had a too small sample size, and the children were in a too narrow age range to further assess the differences in reporting at different ages.

It was interesting to see that adults reported slightly more psychological aggression than the children perceived at endline, even though the difference was not statistically significant. The item that differed most on this subscale at endline was if the caregiver had threatened to kick the child out of the house, where none of the children reported it after the intervention, but 17 percent of the parents said they had done it. This may indicate that the caregivers sometimes made threats that children did not recognize, or that the children did not remember or process it in such a way to report it. This does not fit completely with a systematic review on informant discrepancies in the reporting of child maltreatment that found that in 6 out of 7 studies children reported more emotional abuse than what their caregivers did(Cooley & Jackson, 2020). More research is needed to investigate this further.

The Cronbach alpha scores of internal consistency are not at a satisfactory high level outside of the children’s score at baseline. A possible reason for this is the low number of questions in our scale or the small sample size. A more thorough examination with more items could give a higher alpha score. It could also be because in our study we measured several dimensions of maltreatment, and that will lead to a lower Cronbach alpha score.

Because of the shorter time frame of this study, the lack of randomization and the limited sample size, one can not be certain of the effect the intervention. If there is sufficient funding in the future, one should therefore plan for a randomized controlled trial with follow-up periods to look at the long-term effect of the program. For this project an RCT was not possible.

An important limitation of this study was its small sample size. This may have led to lack of precision and ultimately identification of behavioral differences within and between the groups. Due to our small sample size, we also restricted analysis of data to descriptive and bivariable analysis, and we have not adjusted for possible confounding factors such as caregiver’s education, sex of adult or child, age, which ICDP parenting group the caregivers were participating in with regards to counsellor characteristics or group characteristics, or household population and composition.

The caregivers that were recruited were already in the Pantawid Program, which only includes poverty stricken households (Kandpal et al., 2016), therefore, the population may not be representative for the average caregiver from the Leyte region of the Philippines. Yet, all eligible caregivers from the selected barangays were included which may have made the study representative at least for the study area and the population living under similar conditions.

A comparison with the other four pre-selected barangays on the intervention waiting list was not done. However, this comparison was done between adults having got the intervention and those waiting for the intervention in the Save the children report (Engebretsen, 2018). The report indicated much larger shift towards reduced use of negative discipline strategies in the intervention arm compared to those on the waiting list. The groups were similar at baseline. This strengthens the robustness of our findings, although we do recognize the need for more rigid designed studies with larger sample sizes.

Some loss to follow-up and missing observations might have introduced selection bias and influenced the results. One could anticipate that those with more problems dropped out from the program and data collection, which could give a somewhat skewed result and give an underestimation of the prevalence of maltreatment. However, our findings were similar to the results from a systematic literature review of child maltreatment in the Philippines (Roche, 2017) that found that 56 percent of parents used harsh physical discipline like hitting with objects. Sanapo and Nakumara also found that 49,7 percent of Filipino sixth graders reported that they were exposed to physical punishment at home (Sanapo & Nakamura, 2011).Our study supports findings from other studies about the presence of harsh discipline towards children in the Philippines, including physical harm. Further, it found improved parent strategies following the ICDP parenting training confirmed by both children and adults.

Caregivers reported in general that they used less harsh discipline strategies than the children reported that they received. We cannot exclude the possibility that this could be because of a social desirability bias, where caregivers want to present their relationship and disciplining strategies in line with what is considered to be socially acceptable in this setting.

The difference in reporting before the intervention, and the lack of difference in reporting after the intervention shows the importance of different perspectives on an intervention to show the true effect the intervention had. If the children still had reported significantly more harsh discipline and maltreatment than the caregivers after the intervention, one should question if the intervention program has been good enough, or if there were items in the questionnaire that had been misunderstood by either the children or the caregivers. And also, if we had only interviewed one of the groups and seen a decrease, it could still hide a lacking effect of the intervention. But when both groups showed a decrease, and also agreement on the amount of maltreatment, it gives stronger evidence of a positive impact of the program.

These findings point towards the need for more research, investment and support for ensuring safer home environments for children in the Philippines. It also shows the importance of getting information about child maltreatment from both child and caregiver informants, and illustrates how they can respond differently to questions about maltreatment and mental health, especially when they are interviewed separately. This demonstrates why it is important to include children’s voices, both in a clinical setting, but also in research, because as shown in this study, their voices and points of view can vary significantly from those of an adult, and can contribute to identifying more child maltreatment in addition to showing an important and often overlooked perspective on the caregiver-child dyad.
